# Multiphase analysis by linkage, quantitative transmission disequilibrium, and measured genotype: systolic blood pressure in complex Mexican American pedigrees

**DOI:** 10.1186/1753-6561-8-S1-S108

**Published:** 2014-06-17

**Authors:** Zhijian Chen, Kuan-Rui Tan, Shelley B Bull

**Affiliations:** 1 Lunenfeld-Tanenbaum Research Institute of Mount Sinai Hospital, 60 Murray Street, Box 18, Toronto, Ontario M5T 3L9, Canada; 2 Dalla Lana School of Public Health, Health Sciences Building, 155 College Street, University of Toronto, Toronto, Ontario M5T 3M7, Canada

## Abstract

We apply a multiphase strategy for pedigree-based genetic analysis of systolic blood pressure data collected in a longitudinal study of large Mexican American pedigrees. In the first phase, we conduct variance-components linkage analysis to identify regions that may harbor quantitative trait loci. In the second phase, we carry out pedigree-based association analysis in a selected region with common and low-frequency variants from genome-wide association studies and whole genome sequencing data. Using sequencing data, we compare approaches to pedigree analysis in a 10 megabase candidate region on chromosome 3 harboring a gene previously identified by a consortium for blood pressure genome-wide association studies. We observe that, as expected, the measured genotype analysis tends to provide larger signals than the quantitative transmission disequilibrium test. We also observe that while linkage signals are contributed by common variants, strong associations are found mainly at rare variants. Multiphase analysis can improve computational efficiency and reduce the multiple testing burden.

## Background

In pedigree-based studies, discovery of genomic regions harboring genetic determinants of quantitative traits such as systolic blood pressure (SBP) has conventionally been conducted using linkage analysis based on identity-by-descent allele sharing. In the genome-wide association studies (GWAS) era of cost-effective high-throughput genotyping technology, the mapping of the genetic basis of complex traits/diseases in human populations has been population-based in unrelated individuals, and largely case-control or cross-sectional in design. With the advent of next-generation sequencing technology, investigators are able to examine each single base pair (bp) and test for association with a trait, but the massive amount of variant information available for analysis can be overwhelming. With the development of techniques for pedigree-based imputation from sequence data on selected pedigree members, pedigree-based analysis of whole genome sequencing data is feasible.

**Figure 1:**
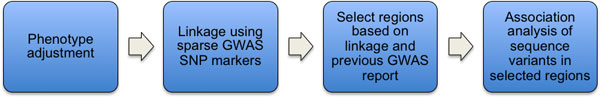
**Workflow for the multiphase linkage and association analysis of a complex pedigree study with GWAS SNP and whole genome sequence data**.

We demonstrate that multiphase analysis in pedigrees can be an efficient strategy for identifying genetic variants underlying a quantitative trait, in which region discovery by linkage analysis of GWAS single-nucleotide polymorphism (SNP) markers with high minor allele frequency (MAF) is followed by region refinement with densely distributed GWAS SNPs and/or fine mapping with sequence variants in identified regions. Using a summary phenotype derived from longitudinal measurements of SBP together with GWAS and whole genome sequencing genotype data from the San Antonio Family Studies (SAFS) as provided by Genetic Analysis Workshop 18 (GAW18), we report pedigree-based linkage and association analysis conducted to identify genetic variants underlying SBP. Our multiphase analyses are carried out in 3 steps, as illustrated by the workflow in Figure 1. First, we obtain a summary phenotype for each individual using the residuals from a censored normal regression model with a random intercept for each pedigree, where the censoring indicator is antihypertensive medication. In the second step, we conduct linkage analysis on chromosome 3 with a sample of GWAS SNP markers (MAF ≥ 5%). We detect linkage at a locus in a region harboring a candidate SNP, rs419076 (bp: 169100886, near *MECOM*, 3q26) identified in a pathway influencing blood pressure and cardiovascular disease risk by the International Consortium for Blood Pressure Genome-Wide Association Studies (ICBP-GWAS) [1]. In step 3, we conduct pedigree-based association analysis using sequence data to fine-map the *MECOM *genomic region.

## Methods

### SAFS pedigree data

From a total of 1389 participants in 20 pedigrees, 932 have SBP measurements at 1 or more study exams for up to 4 exams. Characteristics recorded include sex, year of exam, age at each exam, current use of antihypertensive medications, and current tobacco smoking. GWAS genotypes were assayed in a total of 959 individuals, with a total of 65,519 GWAS SNPs on chromosome 3 available for analysis. Among these individuals, 464 were also sequenced at an average 60 × coverage, resulting in 1,215,399 sequence variants on chromosome 3. For the remaining 495 individuals, the missing genotypes at the sequence variants were imputed using a novel population-based imputation approach [2]. Because the program SOLAR required genotype data, in the focused association analysis following the linkage scan, we used the imputed "best guess" sequence genotypes. Subsequent analyses ignored imputation uncertainty.

### Phenotype adjustment

Antihypertensive medication complicates the analysis of SBP, because patients prescribed medication tend to have elevated underlying SBP values. Based on a novel extension developed by Konigorski et al [3], we treated medication as a right-censoring indicator such that the unmodified SBP for an individual under medication is higher than the observed, and fit a censored normal regression model to the observed SBP measurements for each exam assuming noninformative censoring. In addition, we took into account the between-pedigree variation by incorporating a pedigree-specific random component. Analyzing each of the first 3 visits separately, we included sex, exam-specific age, and smoking status as covariates. Let *Y *be the observed SBP and *Ŷ *be the fitted SBP from the censored model given exam-specific covariates and pedigree-specific random effects. For an individual receiving medication, let $Y^{*}$ be the conditional expectation of the underlying SBP given exam-specific covariates and pedigree-specific random effects and assuming that the underlying unmodified SBP is greater than the observed value, for details see Konigorski et al [3]. We computed residuals at each exam by Y-Ŷ if an individual was not under medication, and by $Y^{*}-Ŷ$ otherwise. The mean of the residuals at exams 1 to 3, denoted by *R*, was then used as an adjusted phenotype for each individual in subsequent stages of linkage and association analysis.

### Variance component linkage analysis

To detect regions with potential loci for SBP, we applied the variance-component linkage method for pedigree-based analysis [4]. In an additive polygenic model, the overall phenotypic covariance matrix Σ for a pedigree of *n *members is partitioned into a locus-specific variance component $\left(\sigma_{\text{qtl}}^{2}\right)$, an additive genetic variance attributable to an unspecified number of remaining loci at unknown locations in the genome $\left(\sigma_{\text{a}}^{2}\right)$, and an environmental variance component $\left(\sigma_{e}^{2}\right)$. Specifically, the phenotypic covariance matrix has the form

$$\Sigma =\Pi \sigma_{\text{qtl}}^{2}+2\Phi \sigma_{\text{a}}^{2}+{\text{I}}_{n}\sigma_{\text{e}}^{2},$$

where the elements of the structuring matrix for the locus-specific variance, Π, are proportions representing the identity-by-descent (IBD) sharing of alleles for each relative pair at this locus; the structuring matrix for the additive genetic variance component, 2Φ, is twice the kinship coefficient matrix; and the matrix for the variance resulting from unshared environmental effects is specified by the identity matrix I*_n_*. To examine the influence of GWAS SNP density on linkage analysis, we sampled 3 sets of SNPs. Initially, a total of 988 SNP markers was randomly sampled from chromosome 3 GWAS SNPs with MAF ≥5%. To allay concerns about adequacy of SNP density, in the second and third samplings, we randomly sampled 1620 and 2999 SNPs, respectively, excluding previously sampled SNPs and using the same MAF criteria. We first performed quantitative genetic analysis to create a suitable null model for each selected marker [4]. Applying the genetic analysis software SOLAR to the sampled GWAS data, we estimated IBD allele sharing for all pairs of relatives in each pedigree, using single-marker estimation to ease computation in the very complex pedigrees. We also performed 2-point rather than multipoint linkage analysis and computed the log of odds (LOD) score for each marker. Regions with LOD >1.2 were considered interesting for subsequent fine mapping analyses. For demonstration purposes, in this paper we focused fine-mapping analyses on the candidate region 165 to 175 megabases (Mb) on chromosome 3.

### Family-based association analysis

In a candidate region on chromosome 3 identified with some evidence for linkage in the sampled GWAS data and previously reported in GWAS meta-analysis [1], we compared the linkage signals to the association analyses implemented in SOLAR: measured genotype (MG) analysis and the quantitative transmission disequilibrium test (QTDT) [5], in which the phenotype, *R*, is modeled as a linear combination of fixed effects (ie, genotype scores) and random effects (ie, polygenic and linkage components). The genotype scores are decomposed into between-family (*b*) and within-family (*w*) components, resulting in fixed-effect model $E\left(R\right)=\mu +\beta_{b}b+\beta_{w}w$. The MG approach estimates regression coefficients with the constraint *β*_b _= *β*_w_. The QTDT approach estimates both *β*_b _and *β*_w_, and tests whether the within-family parameter *β*_w _is significantly different from 0. QTDT reflects the correlation between SNP genotype and phenotype within families and is robust to population stratification effects [5], which can be a concern for MG, but QTDT is less powerful than MG. We computed the IBD allele sharing among pedigree members at each sequence variant in the candidate region, and then performed association tests simultaneously modeling linkage as a variance component based on the IBD sharing estimates. When linkage is present, including the linkage component in the association analysis helps control type I error [6].

## Results and discussion

### Linkage scan

**Figure 2:**
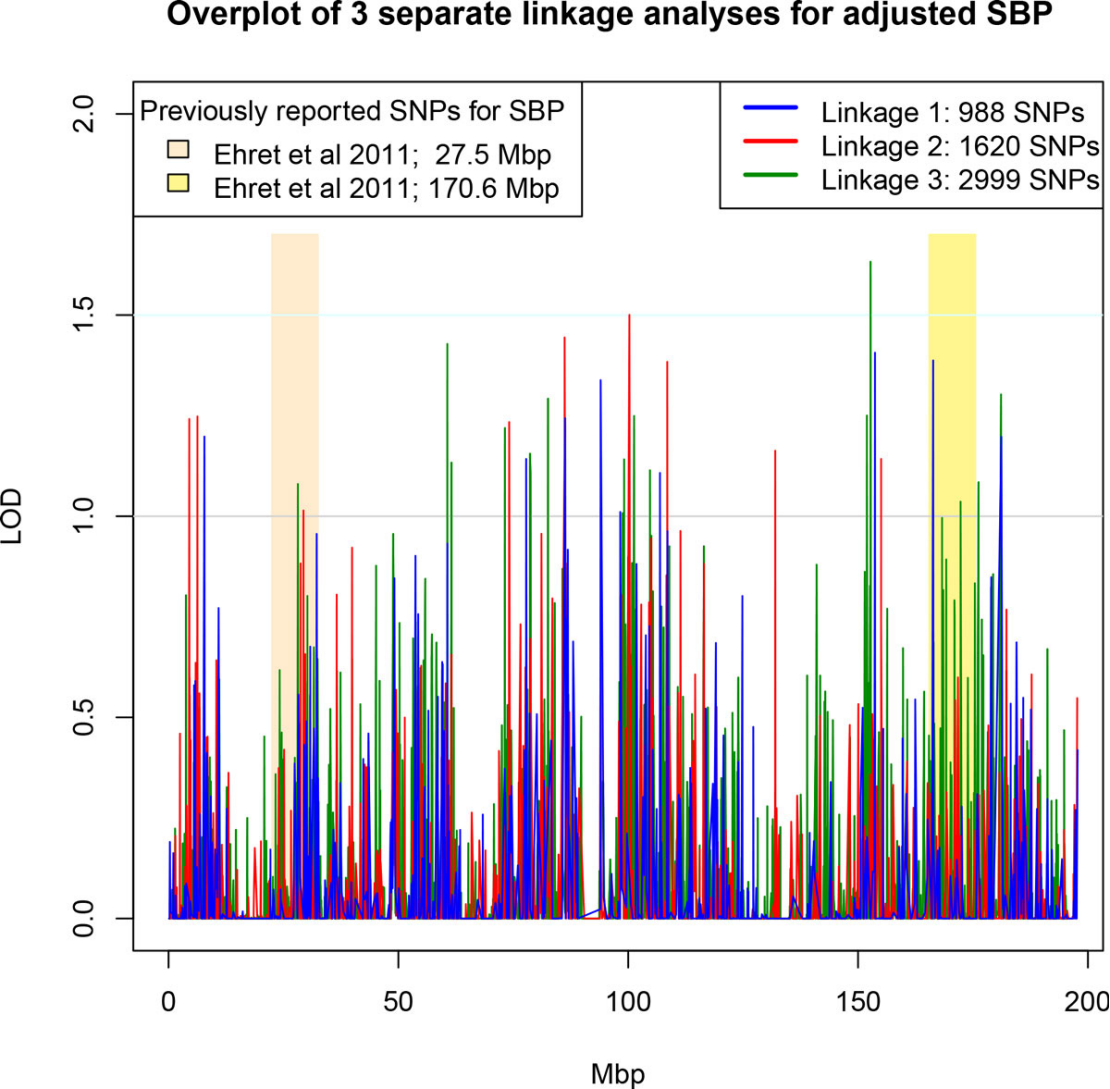
**Two-point linkage analysis for all pedigrees using 3 sets of randomly sampled GWAS SNPs**. The blue, red, and green linkage profiles are for samples of 988, 1620, and 2999 GWAS SNPs, respectively. The beige and yellow regions correspond to 27.5 ± 5 Mb and 170.5 ± 5 Mb, respectively, and the light gray and light blue horizontal lines denote LOD = 1.0 and LOD = 1.5, respectively.

With the first set of 988 GWAS SNPs, evidence for linkage with SBP on chromosome 3 using combined pedigree data was mainly found in 4 regions: 5 to 12 Mb, 47 to 59 Mb, 89 to 115 Mb, and 165 to 175 Mb (Figure 2), with a chromosome-wide maximum LOD score of 1.41. These regions harbor SNP associations identified in a study undertaken by the ICBP-GWAS [1]. In conducting sensitivity analysis using 2 additional sets of randomly sampled GWAS SNPs, we observed multiple linkage peaks in similar regions. The maximum LOD scores for the second and third linkage analyses were 1.50 and 1.63. Although differences in the maximum LOD score among the 3 analyses were not substantial (ie, around 0.23), the maximum LODs did not always correspond to the same region (Table 1). We obtained the names of genes nearest these locations using the annotation report from Nalpathamkalam et al [7].

**Table 1:** Results of 2-point linkage analysis with LOD >1.20, ordered by position, using 3 sets of randomly sampled common GWAS SNPs (MAF ≥0.05) from chromosome 3. LOD scores in bold denote values > 1.35 (column 5).

Marker	Position (Mb)	Position (cM)	SNP set	LOD (>1.2)	Class	Gene
rs304094	4.51744	13.9988	2	1.24	Intergenic	*SUMF1*
rs17044432	6.28766	18.6921	2	1.25	Intergenic	*GRM7*
rs2213215	60.64305	82.6846	**3**	**1.43**	Intronic	*FHIT*
rs9816856	73.18624	100.8683	3	1.22	Intergenic	*PPP4R2*
rs6798130	74.10858	102.7685	2	1.23	Intergenic	*CNTN3*
rs7631179	82.55674	108.3900	3	1.29	Intergenic	*GBE1*
rs13093396	86.16787	108.8700	**2**	**1.44**	Intergenic	*CADM2*
rs9860570	86.28946	108.9715	1	1.24	Intergenic	*CADM2*
rs1598234	94.00416	110.3673	1	1.34	Intergenic	*NSUN3*
rs11719592	100.25710	111.9176	**2**	**1.50**	Intronic	*TMEM45A*
rs4928048	100.27040	111.9277	3	1.20	Intronic	*TMEM45A*
rs4618204	101.28150	112.7198	3	1.25	Intronic	*TRMT10C*
rs16844883	108.49870	118.6100	**2**	**1.38**	Intergenic	*RETNLB*
rs323629	151.92760	161.0093	3	1.25	Intergenic	*LOC401093*
rs1533913	152.70880	162.0010	**3**	**1.63**	Intergenic	*P2RY1*
rs10935963	153.68720	162.6455	**1**	**1.41**	Intergenic	*ARHGEF26-AS1*
rs11916399	166.34160	168.2915	**1**	**1.39**	Intergenic	ZBBX, MECOM*
rs6809553	181.11490	185.8237	3	1.30	Intergenic	*DNAJC19*

*cM*, centimorgan.

*The linkage signal is also close to the gene *MECOM*.

### Association

**Figure 3:**
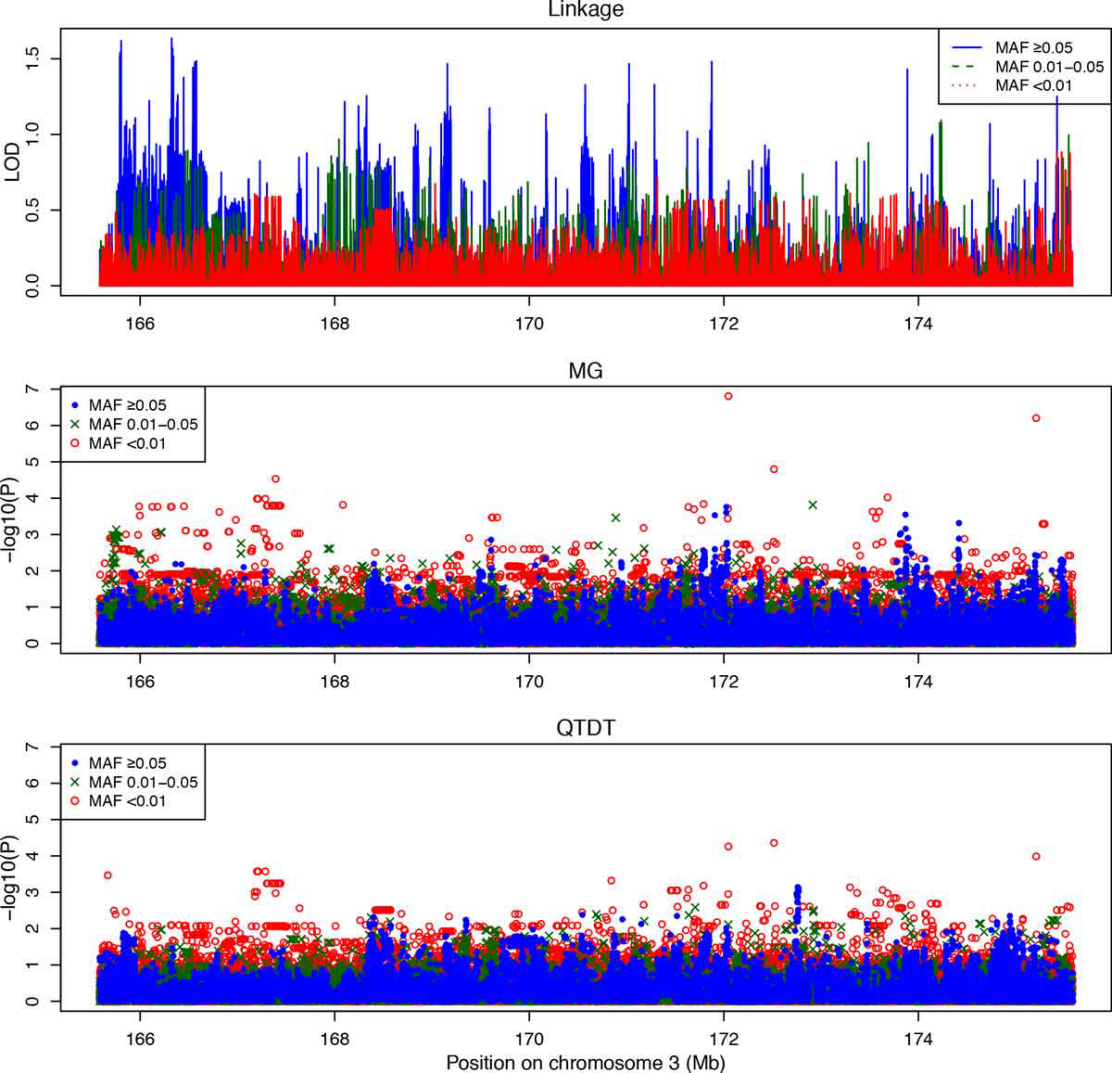
**Linkage and association analysis for a 10-Mb region surrounding the *MECOM *gene using combined pedigrees**. The top, middle, and bottom panels correspond to 2-point linkage, MG association analysis, and QTDT, respectively. Variants with MAF <0.01, 0.01 to 0.05, and ≥0.05 are indicated by red, green, and blue colors, respectively.

Based on our linkage results and prior report by ICBP-GWAS [1], we fine-mapped the 10-Mb chromosomal region (165 to 175 Mb) surrounding the SNP rs419076 in the gene *MECOM *(3q26). Among the 58,651 variants in this region, 20,211 are common (MAF ≥5%), 10,508 are low-frequency (1% to 5% MAF), and 27,932 are rare (MAF <1%). We observed that, as expected, the MG association analysis tended to provide larger signals than the QTDT approach (Figure 3). To assess for global inflation of type I error in the MG and QTDT approaches, we conducted association analysis using the 2999 sample 3 GWAS SNPs. No inflation of type I error was observed in the Q-Q plots for MG, either with or without a linkage variance component. However, the observed type I error rate from the QTDT approach appeared to be slightly deflated, particularly when linkage was included as a variance component (data not shown). This suggests lack of population stratification and is consistent with theory that says the QTDT approach is less powerful than MG for detecting association. Comparing linkage and association results across the 3 variant MAF categories, we observed that linkage signals were contributed by common variants (Figure 3 and Table 2, with the max LOD score observed at bp position 166324439). However, stronger associations were mainly found at rare variants, suggesting the linkage peak may correspond to a haplotype block harboring rare variants underlying blood pressure. The strongest signal was observed at bp position 172046675 with a MG *p *value of $1.56\times 10^{-7}$ (Table 2). Because the analysis was conducted in a candidate region partially selected by independent prior data, we did not require genome-wide significant association, but appropriate criteria in this setting is an open question.

**Table 2:** Top 5 linkage signals and top 5 associations with SBP are indicated in bold in the 165- to 175-Mb region on chromosome 3 (ordered by position)

Position	MAF	LOD score	MG *p *value	QTDT *p *value
165794197	0.403	**1.54**	0.200	0.611
165803609	0.402	**1.54**	0.215	0.543
165804946	0.400	**1.62**	0.257	0.522
166324439	0.118	**1.63**	0.755	0.749
166332595	0.118	**1.57**	0.637	0.695
167201711	0.0021	0.59	**1.04E-04**	**2.66E-04**
167391612	0.0037	0.55	**2.94E-05**	**1.06E-03**
172046675	0.0026	0.06	**1.56E-07**	**5.49E-05**
172516067	0.0021	0.59	**1.58E-05**	**4.37E-05**
175210951	0.0010	0.00	**6.27E-07**	**1.03E-04**

## Conclusions

The main purpose of the proposed multiphase design is to first identify interesting genomic regions for a complex quantitative trait, and then to fine-map those regions in follow-up studies, reducing both the number of tests for association conducted at null variants and the computational processing time. With randomly sampled common GWAS SNP data for large Mexican American pedigrees from SAFS, we identified 4 linkage regions for SBP on chromosome 3. Especially for 2-point linkage, high-density SNP analysis is desirable. In linkage analysis in an identified region, we observed higher LOD scores using imputed sequence data compared to GWAS SNP data, particularly for common variants (Figure 3, *top panel*). In family-based association analysis of sequence variants, however, we observed stronger association signals at rare variants compared to common variants. As is typical in fine-mapping studies, we examined association with sequence variants under linkage peaks obtained from a chromosome-wide scan. Depending on the inherent power in a study, it may be advisable to establish a fairly liberal criterion for identification of linkage regions. Although the linkage strategy we used reduces the multiple testing burden in phase 2, it may miss regions of interest that would have been detected by a GWAS association analysis. For purposes of comparison, albeit in a single data set, we examined the results from a complete, dense GWAS scan of chromosome 3 that used mixed models to account for the pedigree structure [8]. We observed that both strategies identified regions near 150 Mb and 175 Mb using a linkage criterion of LOD >1.0 and a GWAS criterion of *p *<10^−5^; the chromosome-wide maxima near 150 Mb agreed quite well. Our linkage scan also identified regions at other locations, including those near 10, 27, and 100 Mb, that would have required more liberal GWAS criteria for identification.

## Competing interests

The authors declare that they have no competing interests.

## Authors' contributions

ZC designed the overall study, conducted the sequence variant analyses, and drafted the manuscript. KRT conducted the chromosome-wide linkage scan using sampled GWAS markers. SBB conceived the study, participated in its design and conduct, and helped to revise the manuscript. All authors read and approved the final manuscript.
